# Prompt-Gamma Activation Analysis

**DOI:** 10.6028/jres.098.009

**Published:** 1993

**Authors:** Richard M. Lindstrom

**Affiliations:** National Institute of Standards and Technology, Gaithersburg, MD 20899

**Keywords:** activation analysis, cold neutron beams, elemental analysis, neutron capture gamma rays, nuclear analytical methods, prompt gamma-rays

## Abstract

A permanent, full-time instrument for prompt-gamma activation analysis is nearing completion as part of the Cold Neutron Research Facility (CNRF). The design of the analytical system has been optimized for high gamma detection efficiency and low background, particularly for hydrogen. Because of the purity of the neutron beam, shielding requirements are modest and the scatter-capture background is low. As a result of a compact sample-detector geometry, the sensitivity (counting rate per gram of analyte) is a factor of four better than the existing Maryland-NIST thermal-neutron instrument at this reactor. Hydrogen backgrounds of a few micrograms have already been achieved, which promises to be of value in numerous applications where quantitative nondestructive analysis of small quantities of hydrogen in materials is necessary.

## 1. Introduction

The nuclei of some elements of a sample placed in a field of neutrons absorb neutrons and are transformed to an isotope of higher mass number. Conventional neutron activation analysis employs the radiations emitted during the decay of radioactive products for elemental analysis. Some elements do not produce radioactive capture products, but do emit prompt gamma rays at the time of neutron capture. If the sample is placed in an external neutron beam from a reactor and viewed by a high-resolution gamma-ray spectrometer, these gamma rays allow qualitative identification and quantitative analysis of the neutron-capturing elements present in the sample.

**Fig. 1:**
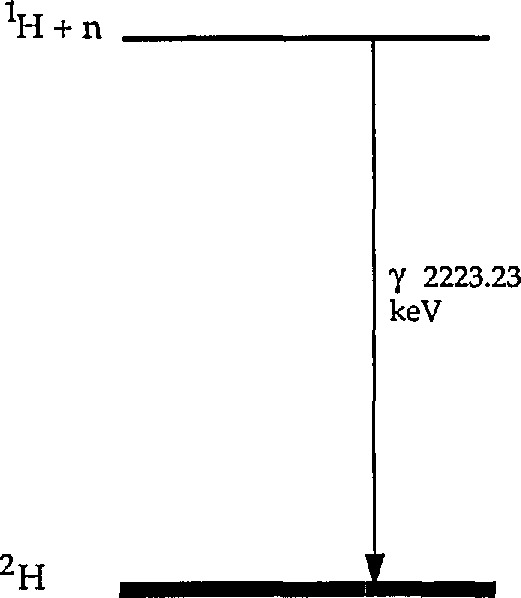
Energy level diagram of the *A* = 2 system.

As the simplest example, ^1^H captures a neutron to produce an excited nuclear state of deuterium (Fig. 1). The energy of this state is precisely determined through the Einstein relation by the difference between the masses of the separated neutron and proton and that of the ground state of the deuteron. For a slow neutron this energy is 2224.6 keV. The only available deexcitation mode of this compound nucleus is by the emission of a gamma ray of 2223.23 keV energy, the balance of the reaction energy being carried off as recoil by the deuteron. The presence of a gamma ray of this energy in the spectrum of a specimen during neutron irradiation indicates the presence of hydrogen in the sample, and the intensity of this gamma ray relative to a standard is a quantitative measure of the amount of hydrogen present. This analytical technique has been given a number of names, most often neutron-capture prompt-gamma-ray activation analysis, which we abbreviate as PGAA. Cold neutrons offer substantially improved analytical sensitivity over thermal neutron beams.

## 2. Principles

### 2.1 Experimental

**Fig. 2:**
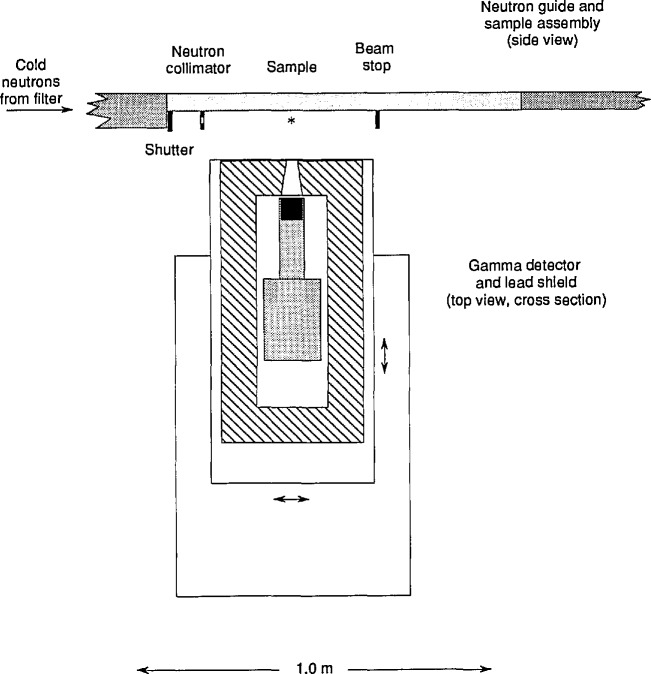
Schematic of the PGAA apparatus.

The apparatus is conceptually simple (Fig. 2): A collimateci beam of neutrons is extracted from the reactor and the sample inserted into the beam. A germanium detector, coupled to a multichannel pulse height analyzer and computer, measures the energy and intensity of the prompt gamma radiation emitted. The apparatus is completed by a beam stop to absorb the neutrons which are not absorbed by the sample, and the shielding necessary to protect the detector and the experimenters from stray gamma rays and neutrons.

### 2.2 Applicability

The use of neutron-capture gamma rays as a method of elemental analysis was introduced many years ago [1–3]. With the development of large, high-resolution gamma-ray detectors in the past decade, PGAA has taken its place as a complementary technique alongside conventional neutron activation analysis [4,5]. This method is particularly useful for determining nondestructively elements which absorb neutrons but do not produce radioactive products. The PGAA method analyzes the entire sample, including any substrate or container by which it is supported in the beam. The values of the nuclear parameters and the abundances of the elements in common materials are such that PGAA finds its greatest applicability in the determination of nonmetals that form the major and minor elements of geological and biological materials (H, C, N, Si, P, S), or trace elements with high thermal capture cross sections (B, Cd, Gd) that are not readily determinable by other techniques.

PGAA has been used alone to measure up to 21 elements in standard rocks [6,7], and in combination with conventional instrumental neutron activation analysis (INAA) to measure as many as 48 elements in coal without chemical separation [8]. These two complementary techniques have been extensively used in the study of natural and man-made atmospheric aerosols [9]. Two bibliographies of PGAA applications have been compiled [10,11].

Partly because of the need for continuing access to a reactor neutron beam, application of PGAA as a routine method of elemental analysis has been pursued to date at only a few laboratories on a full-time basis (for reviews see [12,13]). Because of lower neutron fluence rate and (usually) lower gamma-ray detection efficiency, the sensitivity of the method for most elements is two to three orders of magnitude worse than INAA, which limits most routine applications to the determination of the above mentioned elements. Irradiation times of at least several hours are required for most multielement analysis, hence the throughput is low because only one sample can be irradiated and measured at a time.

The sensitivity of PGAA for a given element, expressed in counts·s^−1^g^−1^, is given by

$$S=\frac{N_{A}\;I\;\sigma \;\phi \;\Gamma \in (E)}{M},$$ (1)

where

*S* = sensitivity, counts·s^−1^g^−1^
*N*_A_ =Avogadro’s number
*I* = fractional abundance of the capturing isotope
*σ* = neutron capture cross section, cm^2^
*ϕ* = neutron fluence rate, cm^−2^s^−1^
*Γ* = gamma ray yield, photons per capture
*ϵ*(*E*)=gamma ray detection efficiency at energy *E*, counts/photon
*M* = atomic weight

The useful detection limit in practice is set by the sensitivity, the counting precision required, the blank (signal in the absence of a sample), and the peaked and continuum background caused by all components of the sample.

### 2.3 Sample Considerations

For a successful PGAA measurement, the sample must be large enough for the analyte to give a usefully strong signal, and small enough that the total capture rate is not too high for the detector and that neutron and gamma-ray scattering and absorption within the sample gives acceptably small errors. For many materials the optimum sample size lies between 0.1 and 10 g. Samples with special geometry such as entire silicon wafers can be accommodated. Ready access to the sample position may make feasible the nondestructive analysis at low temperature of volatile materials such as solid cometary samples [14].

In the analysis of plant and animal tissue, both detection limits and accuracy of PGAA measurements are often determined by the amount of hydrogen in the sample. The strong hydrogen capture gamma ray at 2223.2 keV is accompanied by a high Compton continuum, which makes the detection limits of other elements below 1995 keV poorer than they would otherwise be. Active Compton suppression can reduce this baseline substantially, but not eliminate it. Because of neutron scattering in the sample, a high concentration of hydrogen in the analytical matrix may lead to either larger or smaller signals per gram of the elements of interest [15,16]. This source of bias is minimized for spherical or near-spherical samples [17,18].

## 3. PGAA at the CNRF

### 3.1 Cold Neutrons

For chemical analysis, the ideal neutron field has the largest possible number of activating particles (slow neutrons) per second per unit area at the sample, and the smallest possible number of interfering particles (fast neutrons and background gamma rays) at the detector. A narrow beam is desirable so that the gamma-ray detector can be moved near the sample and the size of the shielding may be minimized. The beam need not be parallel, but its intensity should be uniform across the target.

A guided beam of cold neutrons meets these requirements very well [19]. Cold neutrons have been applied to PGAA in only a few laboratories to date [20–23], though several instruments are under construction or active planning [24–26]. As Maier-Leibnitz pointed out long ago, the reduction in background by use of a high-quality beam is more important for analytical purposes than is an increase in the capture rate [19]. Henkelmann and Born have demonstrated this by collecting spectra with very low continuum background using a curved neutron guide at a cold source [23]. Experience has shown that with purely slow neutrons a neutron collimator and beam stop can be simple, lightweight, and compact; merely thin slabs of metallic ^6^Li or a ^6^Li compound without bulky thermalizing material or heavy gamma absorbers [27,21]. In consequence of the low neutron and gamma-ray background, the gamma detector can be placed close to the sample where the detection efficiency is high.

With the high gamma-ray detection efficiency possible with cold neutron beams, a practical limitation on the analytical usefulness of PGAA will be the ability to collect data at high counting rates without distortion. Recent advances in fast amplifier design [28] make it likely that the collection time for electrons in germanium may become the rate-limiting process. With large Ge detectors coupled with compact high-Z gating detectors, Ge-Ge coincidence counting may be done with profit. Multiparameter counting offers a good solution to the problem of interfering lines in a crowded spectrum.

The capture rate is also higher with cold than with thermal beams. Since capture cross sections for most target nuclei are inversely proportional to the neutron velocity, a neutron at 30 K is three times as effective as one at 300 K. Multiple reflections of the neutrons in a straight guide ensure that the intensity of the beam is more uniform across the sample than in most thermal irradiation facilities.

### 3.2 The CNRF Instrument

In the construction of the PGAA instrument at the CNRF, experience gained with cold neutrons at the German Nuclear Research Center in Jülich [21] and with over a decade’s operation of the Maryland-NIST thermal instrument at the NBSR [6] has been incorporated to give high efficiency, low background, and facile operation [23]. The NIST instrument is installed on neutron guide NG-7 in the CNRF hall (see Fig. 7 of Prask et al., p. 11 this issue). A filter of 152 mm of single-crystal Bi and 127 mm of Be, both at liquid nitrogen temperature, is installed in the guide 3.1 m upstream from the PGAA sample position.

The apparatus is shown in Fig. 2. The lower 50 × 45 mm of the 50 × 110 mm NG-7 guide is extracted into air through a window of magnesium 0.25 mm thick. (The upper 50 × 50 mm beam continues past the PGAA station another 2 m to the velocity selector of the 30 m SANS instrument.) A translating shutter of ^6^Li glass [30] 15 mm thick just behind the window admits the neutrons to the sample. The 10 mm thick glass slab which forms the bottom of the continuing guide is capped with 15 mm of ^6^Li glass to avoid background from neutron scattering and capture. The neutron fluence rate was measured with 25 µm Au to be 1.5 × 10^8^ cm^−2^s^−1^ (thermal equivalent: using σ = σ_th_ = 98.65 b) at a reactor power of 20 MW.

The 1 m section of neutron guide adjacent to the PGAA station is made of boron-free silicate glass in order to avoid generating large amounts of ^10^B capture gamma radiation from neutron leakage due to imperfections and misalignment of the guide. To reduce the neutron background, the outside of this guide section is covered with a ^6^LiF-graphite paint [31]. A plate of fused ^6^Li_2_CO_3_ [32] with a 20 mm aperture, placed just behind the shutter, collimates the neutron beam to a size not much larger than a typical sample. A beam stop of ^6^Li glass is placed behind the sample. Secondary fast neutrons generated in the collimator and beam stop by reactions of the fast tritons from ^6^Li(n,α)^3^H [33] have not yet been troublesome. Hydrogen-containing materials have been avoided to the maximum extent possible in the vicinity of the sample and detector. Samples are held in the beam in envelopes of 25 µm Teflon^1^ FEP held by taut strings of 200 µm Teflon PFA between the prongs of a supporting fork. The volume between the neutron collimator and the beam stop assembly can be flooded with He at atmospheric pressure in a Teflon tent to reduce air scatter and reduce the hydrogen and nitrogen background.

A Ge gamma-ray detector (27% relative efficiency, 1.7 keV resolution) views the sample through a ^6^Li-glass window along an axis at right angles to the neutron beam. The field of view of the detector is collimated so as to view chiefly the sample. Environmental gamma radiation is reduced by shielding the detector with at least 100 mm of lead in all directions. The lead in turn is shielded from stray neutrons (which produce 7 MeV Pb capture gamma rays) with sheets of Boral. The shielded detector is carried on a table, the top of which is adjustable vertically and parallel to the beam with leadscrews. The table rolls on a track perpendicular to the beam to adjust the counting distance, which can be as close as 200 mm from the sample to the front face of the detector. Experiments are continually under way to optimize the shielding in the vicinity of the sample-detector assembly.

Gamma-ray spectra are acquired with a 16384-channel analog-digital converter (Canberra Nuclear Data ND581 ADC) coupled to a multichannel pulse height analyzer (ND556 AIM). The AIM is controlled over Ethernet by Nuclear Data acquisition and display software on a VAX-station 3100, which in turn communicates for data analysis with a Micro VAX 3400 and numerous other computers and terminals on the building-wide Ethernet [34].

### 3.3 Applications

Several measurements have been made in the short time that this cold-neutron instrument has been in operation [23]. Sensitivities of a number of elements were compared with those of the Maryland-NIST thermal PGAA instrument. At the same reactor power, sensitivities for most elements are a factor of four to six better with the cold-neutron apparatus. Expected detection limits with the CNRF instrument are given in Table 1, which are extrapolated from measured detection limits in biological and geological materials with the existing thermal PGAA instrument at NIST [13].

The first measurement with cold neutron PGAA in the CNRF was the determination of hydrogen in a sample of C_60_ fullerene “buckyballs” intended for neutron scattering studies. Cold-neutron PGAA found a hydrogen concentration of 0.92 ± 0.09 wt%, which is too high for satisfactory scattering measurements. After repurification of the material, hydrogen was measured again. A 600 mg sample, contained in the aluminum sample holder intended for the scattering measurements, was irradiated in the PGAA beam for 100 min live time while surrounded with a flowing atmosphere of He contained in a Teflon tent. A clear H peak was visible at more than ten times the intensity of a blank sample of spectroscopic grade graphite. The sample was found to contain 0.077 ±0.014 wt *%* H, which was adequately low for the neutron scattering measurements. Numerous samples of pure and substituted fullerenes have been analyzed (e.g., [35]).

In other work, hydrogen was measured in a Pr_2_CuO_4_ superconductor analog; a 1 h irradiation gave a concentration of [H] = 0.017 ±0.010 wt %. Hydrogen was sought in a 50 nm borophosphosili-cate glass film on a quarter of a 10 cm silicon wafer; an upper limit of 2 µg/cm^2^ was found.

## 4. Future Plans

Future enhancements to the counting system will include a redesigned sample positioning and shielding assembly, a Compton suppressor, an automatic sample changer, and a second detector system which will permit γ-γ coincidence measurements. A new cold source under design is predicted to increase the neutron fluence rate by a factor of about five. The difficulties associated with working adjacent to the upper guide — restricted space and Si capture background—may be ameliorated in the future by deflecting the PGAA beam away from the guide. Additional improvement in sensitivity is possible since neutron optics may be used to focus cold neutrons onto a small area. Gains of an order of magnitude in fluence rate may be obtainable by these techniques [36–38].

## Figures and Tables

**Table 1:** Expected interference-free detection limits for neutron-capture prompt gamma-ray activation analysis^a^

Element	Dct. limit, µg	E_γ_,keV
Hydrogen	2	2223
Boron	0.003	478
Carbon	4000	1262, 4945
Nitrogen	400	1885, 5298
Fluorine	500	583, 1634(D)^b^
Sodium	7	472, 869
Magnesium	200	585, 1809
Aluminum	50	1779(D), 7724
Phosphorus	200	637, 1072
Sulfur	30	840, 2379
Chlorine	1	517, 786
Potassium	10	770, 7771
Calcium	60	519, 1943
Titanium	4	342, 1381
Vanadium	4	1434(D)
Chromium	15	749, 834
Manganese	3	847(D), 1811(D)
Iron	30	352, 7631
Cobalt	1	230, 556
Nickel	20	283, 465
Copper	1	159, 278
Zinc	70	115, 1077
Arsenic	10	164
Selenium	4	239
Bromine	10	195, 244
Strontium	40	898, 1836
Molydenum	15	720, 778
Silver	3	192, 236
Cadmium	0.01	559, 651
Indium	0.5	162, 186
Barium	10	627, 818
Neodymium	1	619, 697
Samarium	0.003	333, 439
Gadolinium	0.002	182, 1186
Gold	3	215
Mercury	0.15	368
Lead	4000	7368

a Assumptions: 24 h irradiation with NBSR at 20 MW.

b (D) signifies a decay gamma ray, not prompt.

## Footnotes

1 Certain commercial equipment, instruments, or materials are identified in this paper to specify adequately the experimental procedure. Such identification does not imply recommendation or endorsement by the National Institute of Standards and Technology, nor does it imply that the materials or equipment identified are necessarily the best available for the purpose.
